# Myocardial conditioning techniques in off-pump coronary artery bypass grafting

**DOI:** 10.1186/s13019-014-0204-7

**Published:** 2015-01-20

**Authors:** Marco Moscarelli, Prakash P Punjabi, Gamov I Miroslav, Paolo Del Sarto, Francesca Fiorentino, Gianni D Angelini

**Affiliations:** NHLI, Hammersmith Hospital, Imperial College London, London, UK; Fondazione Monasterio, Ospedale Pasquinucci, Massa, Italy; Bristol Heart Institute, University of Bristol, Bristol, UK

**Keywords:** Off-pump coronary artery bypass, Ischemic preconditioning, Volatile anaesthetics

## Abstract

Off-pump coronary artery bypass surgery by avoiding cardioplegic arrest seems to reduce the risk of ischemic myocardial injury. However, even short-term regional ischemic periods, hemodynamic instability and arrhythmias associated with the procedure can be responsible for myocardial damage. Conditioning, a potential cardio-protective tool during on-pump cardiac surgery, has hardly been investigated in the context of off-pump surgery. There are virtually no large trials on remote ischemic preconditioning and the majority of reports have focused on central ischemic conditioning. Similarly, volatile anesthetic agents with conditioning effect like ischemic preconditioning have been shown to reduce cardiac injury during on-pump procedures but have not been validated in the off-pump scenario. Here, we review the available evidence on myocardial conditioning, either with ischemia/reperfusion or volatile anesthetic agents in patients undergoing off-pump coronary artery surgery.

## Review

Conditioning is an umbrella definition that consists of pre-conditioning, per-conditioning and post-conditioning [1]. Conditioning can be elicited remotely (e.g. at the level of a limb) [2] or centrally (e.g. at the level of the heart) [3]. Stimulus normally consists of an ischemic period followed by reperfusion [4], but other triggers like pain-stimulus [5], hyperbaric oxygen [6] and most importantly methods such as volatile anesthetics [7] have been advocated as being equally effective. Conditioning has been experimented in cardiac surgery mainly in the form of remote ischemic preconditioning (RIPC) and the majority of the largest trials have been in on-pump CABG patients (ONCAB) [8] or during valve surgery [9]. RIPC seems to lowers troponin release in patients undergoing ONCAB (‘proof of concept’), but still unclear is if this leads to any better clinical outcome. RIP Heart-Study will recruit over 2000 on-pump patients and will include as primary outcomes all-cause mortality, non-fatal MI, any new stroke and/or acute renal failure [10] and the ERICCA [11] trial will establish if there would be better clinical outcome in on-pump high-risk patients. However, it is still an open question whether conditioning has potential benefits in patients undergoing OPCAB in terms of lowering troponin release and improving clinical outcomes. Ischemia during OPCAB can happen in 10% of cases [12] with ST segment elevation in up to 40% of patients [13]. Although the use of intra-coronary shunts can reduce the ischemic time, even short-term regional ischemic periods or heart manipulations can result in myocardial injury [14], and subsequent arrhythmias and/or hemodynamic instability which can lead even to conversion to ONCAB [15].

Several reports on the use of volatile anesthetics cardio-protective agents in ONCAB surgery have suggested a similar ischemic preconditioning effect. However, only small trials have investigated the effect of anaesthetic preconditioning in OPCAB. The use of alternative methods such as adenosine, hyperbaric oxygen or temperature in OPCAB is also yet to be validated. Optimizing protection of the heart from ischemia/reperfusion injury (I/R) during OPCAB is, therefore, a worthwhile goal.

### Ischemic conditioning in OPCAB

Although there are evidences supporting the benefits of ischemic preconditioning (IP) in terms of cardio-protection, its adoption in OPCAB has been relatively limited (Table 1) with just few studies published so far.

**Table 1:** **Studies including ischemic conditioning**

**Author, date, journal and country study type**	**Patient group**	**Type of conditioning**	**Outcomes**	**Key results**	**Comments**
Joung et al. (*2013*) Korean J Anestesiol, Korea [16] Prospective controlled randomized trial	Seventy OPCAB	RIPC 4 cycles of 5 min ischemia and 5 min of reperfusion before coronary artery anastomoses	Six cognitive function test day 1 after surgery	Post-operative cognitive dysfunction was 28.6% (10 pts) and 31.4% (11 pts) in RIPC and Control group respectively	RIPC did not reduce incidence of post-op cognitive dysfunction after OPCABG during the immediate post-op period
	35 RIPC				
	35 Control				
Forouzannia et al. (*2013*) J The Univ Heart Ctr, Iran [17] Prospective controlled randomized trial	Sixty OPCAB	Adenosine.	Post-op EF	IP and adenosine did not elicit statistically significant EF preservation compared to the control group	No difference found in post-op EF and enzymes release in between groups. Incidence of arrhythmias was higher in the IP group but did not reach statistical significance
	20 Adenosine	IP induced with twice 2 min LAD occlusion followed 3 min reperfusion before the first anastomosis	Arrhythmias		
	20 IP		Troponin/CK-MB		
	20 Control				
Hong et al. (*2012*) Circulation Journal, Japan [18] Prospective controlled randomized trial	Seventy OPCAB	Lower limb 4 cycles of 5 min ischemia and 5 min of reperfusion before anastomoses (RIPC) and after anastomoses (RIPostC)	Troponin release	RIPC + RIPostC significantly reduced postoperative serum troponin I levels	RIPC + RIPostC decreased postoperative myocardial enzyme elevation by almost half postoperatively in patients undergoing OPCAB
	35 RIPC + RIPpostC				
	35 Control				
Hong et al. (*2010*)] Anaesth Intensive Care, Korea [19] Prospective randomized controlled trial	130 OPCAB	Upper limb 4 cycles of 5 min ischemia and 5 min of reperfusion after anesthesia	Troponin release	Troponin release was lower in the RIPC group but was not statistically significant	RIPC did not reduce significantly post-operative myocardial enzyme release
	65 RIPC				
	65 Control				
Succi et al. (*2010*) Arq Bras Cardiol, Brasil [20] Prospective controlled randomized trial	Forty OPCAB	IP induced with twice 1 min LAD occlusion followed 2 min reperfusion before the anastomosis	Intra-op EF (measured pulsed Doppler of the descending thoracic aorta)	Acceleration of the aortic blood flow with no differences in between groups; IP group maintained left ventricular contractility during the entire procedure while the control group presented significant reduction in left ventricular contractility	IP prevented the decrease in left ventricular contractility during off-pump myocardial revascularization surgery
	0 IP				
	20 Control				
Drenger et al. (*2008*) Journal of Cardiothoracic and Vascular Anesthesia, Israel [21] Prospective controlled randomized trial	Twenty five OPCAB	IP induced with single 5 min LAD occlusion followed by 5 min reperfusion 1.6% ENF started 15 min before LAD occlusion	Myocardial metabolism	Lactate production in the ENF group decreased significantly compared with control and IP groups. Oxygen utilization in the control was 44% higher than the other two groups. Early recovery of anterior wall hypokinesis in both study group	Application of methods such as IP or volatile anesthesia appeared to reduce the metabolic deficit
	8 Control				
	9 IP				
	8 Enflurane				
Wu et al. (*2003*) Journal of Cardiothoracic and Vascular Anesthesia, Finland [22] Prospective controlled randomized trial	Thirty two OPCAB	IP induced with twice 2 min LAD occlusion followed 3 min reperfusion before the first anastomosis	Incidence of post-operative arrhythmias	IP suppressed the HR elevation during the time of myocardial ischemia and reperfusion and significantly reduced the incidence of VT after surgery. Incidence of SVT during 2 to 24 hours after surgery was lower in the IP patients but incidence of SVES, VES, and AF were similar between the 2 groups	Arrhythmia was a common phenomenon during and after OPCAB procedure; IP protocol significantly suppressed HR elevation, episodes of SVT, and incidence of VT after surgery but incidence of post-op AF was similar in between groups
	16 IP				
	16 Control				
Doi et al. (*2003*) Jpn J Thorac Cardiovasc Surg, Japan [23] Prospective observational study	Forty-five OPCAB (MIDCAB)	IP induced with 5 min vessel occlusion followed 5 min reperfusion before anastomosis	phiL/phiT, QT, JT dispersions before, during and after IP and during and after coronary anastomosis	Anisotropy was exaggerated during the 5-minute coronary occlusion; during anastomosis, conduction velocities were decreased, but showed no further deterioration; QT and JT dispersions were improved by reperfusion	Anisotropy and dispersions were minimized after IP, therefore IP demonstrated antiarrhythmic protective effects on the human myocardium
Laurikka et al. (*2003*) Chest, Finland [14] Prospective controlled randomized trial	Thirty-two OPCAB	IP induced with cycle of twice 2 min LAD occlusion followed 3 min reperfusion before the first anastomosis	Myocardial performance	IP group had complete recovery of mean after the operation; in the control subjects, mean SVI showed a significant reduction postoperatively	IP tended to decrease the immediate myocardial enzyme release, prohibited the postoperative increase in HR, and enhanced the recovery of SVI
	16 IP				
	16 Control				
Matsumoto et al. (*2001*) Kyobu Geka, Japan [24] Retrospective observational study	Forty-three OPCAB	IP induced with twice 5 min vessel occlusion followed 5 min reperfusion before anastomosis Allopurinol preoperatively and nicorandil intraoperatively;	Myocardial tissue oxygen saturation	Troponin level was statistically significant lower in the IP group On day 1 post op, the increase in the mean HR was also significantly lower in the IP group Significant amelioration of post-ischemic recovery in the IP + pharmacological preconditioning	Concomitant use of IP and KATP opener, oxidative radical scavenger both ameliorated cardiac dysfunction during ischemia in anastomotic occlusion of the coronary artery and improved the post-ischemic functional recovery
	12 IP				
	29 IP+pharmacological		Post-ischemic functional recovery		
van Aarnhem et al. (*1999*) Eur J Cardiothorac Surg, The Netherlands [12] Retrospective observational study	Two-hundred OPCAB	IP induced with 5 min of local coronary artery occlusion and 5 min of reperfusion before anastomosis	Ischemia during temporary coronary artery occlusions	Ischemia (defined as defined as > 1 mm S-T segment) occurred during 35 (10%) temporary coronary artery occlusions	Temporary segmental occlusion was safe before anastomosis in OPCAB; shunts were used in critical ischemia Ischemic dysfunction was precipitated by the 5-min LAD occlusion, as shown by the increase in LVWMS and PA pressure. However, a 5-min coronary occlusion and the resulting ischemia did not alter regional LV systolic function during subsequent ischemia
				There were no perioperative MI/no conversion to ONCAB LVWMS decreased significantly after first cycle but improved after IP No significant differences in pulmonary artery pressures were after IP and during anastomosis	
Malkowski (*1998*) J Am Coll Cardiol (USA) [13] Prospective observational study	Seventeen OPCAB (MIDCAB)	IP induced with 5 min of local coronary artery occlusion and 5 min of reperfusion	LVWMS		
			PA systolic and diastolic pressure		

*AF*: Atrial fibrillation; *IP*: Ischemic preconditioning; *I/R*: Ischemia reperfusion; *LAD*: Left anterior descending artery; *LVWMS*: Left ventricle wall motion score; *MIDCAB*: Minimally invasive direct coronary artery bypass grafting; *ONCAB*: On-pump CABG; *OPCAB*: Off-pump CABG; *phiL/phiT*: Ratio of longitudinal to transverse conduction velocity *PostC*: Postconditioning; *RIPC*: Ischemic remote preconditioning; *SVI*: Stroke volume index; *SS*: Sevoflurane.

Malkowsky et al. [13] induced IP with 5 minutes of local coronary artery occlusion and 5 minutes of reperfusion in a series of 17 single vessel OPCAB patients. Left anterior descending artery (LAD) occlusion/reperfusion increased left ventricle wall motion score (LVWMS) and pulmonary pressure (PA). However, subsequent ischemia during the construction of the anastomosis did not alter the regional LV systolic function. van Aarnhem et al. [12] retrospectively reviewed a cohort of 200 OPCAB patients in whom ischemic preconditioning was used before anastomosis by occluding the vessel for 5 minutes and then allowing 5 minutes of reperfusion. They reported a 10% incidence of-intra operative ischemia (defined as 1 mm ST segment elevation) but no perioperative myocardial injury. Intra–coronary shunts were used in case of critical ischemia and no conversion to ONCAB was observed. Laurikka et al. [14] showed that IP, induced with two cycles of 2 minutes left anterior descending artery occlusion (LAD) followed by 3 minutes of reperfusion before the first coronary artery anastomosis, decreased the immediate myocardial enzyme release, reduced the post-operative increase in HR, and enhanced the recovery of volume index after surgery. Doy et al. demonstrated that a single cycle of 5 minutes of central I/R attenuated ischemia-induced electrophysiological changes in patients undergoing MIDCAB [23]. Wu et al. [22] reported that IP, induced by to cycles of 2 minutes occlusion of LAD followed by reperfusion, led to a positive suppression of HR and reduced the incidence of supra-ventricular and ventricular arrhythmias, although the incidence of post-operative atrial fibrillation (AF) remained similar between preconditioned and non-preconditioned groups. Drengen et al. [21] compared prospectively a cohort of patients preconditioned with a single cycle of 5 minutes of LAD occlusion followed by 5 minutes of reperfusion and a cohort preconditioned with 1.6% enflurane with a non-preconditioned group. They reported a significant reduction of metabolic deficit in both the IP and volatile groups compared to the non-preconditioned group [21]. Hong et al. [19] carried out a prospective controlled randomized trial on RIPC in patients undergoing OPCAB. Remote ischemic preconditioning was elicited with 4 cycles of 5 min upper limb ischemia and 5 min of reperfusion after anesthesia. Troponin release was lower in the RIPC group but did not reach statistical significance (p = 0.172) [19]. Succi et al. [20] reported that IP, induced only by 1 minutes of LAD occlusion followed by 2 minutes of reperfusion before anastomosis prevented decrease in left ventricular contractility Hong et al. [18] used both RIPC and postconditioning (PostC) in a cohort of 35 OPCAB patients. Ischemic remote preconditioning and PostC were elicited with 4 cycles of 5 minutes of ischemia and 5 minutes of reperfusion of the lower limb before and after anastomosis. They reported a significant decrease in post-operative myocardial enzymes. Forouzinna et al. [17] prospectively compared patients preconditioned either with adenosine or with 2 cycles of 2 minutes of LAD occlusion followed by reperfusion with a non preconditioned control group. They reported no differences in terms of post-operative EF and enzyme release but the incidence of post-operative AF was higher in the IP group although it did not reach statistical significance. A recent pilot study by Jung et al. focusing on neurological outcome did not show any benefits of RIPC in patients undergoing OPCAB in terms of cognitive outcomes [16].

### Volatile anesthetic agents conditioning in OPCAB

Several reports investigating the potential use of volatile anesthetics as preconditioning agents in OPCAB (Table 2) have reported rather different results. Sevoflurane was found to reduce troponin release in three trials [25-27], although Orriach et al. used sevoflurane as well as post-conditioning agent, extending its administration during the first post-operative hours [25]. No differences in troponin release were reported by other authors when sevoflurane was compared to desflurane and propofol [28], to propofol in a remifentanil**-**based anesthesia regime [29,30] and to isoflurane alone [31,32]. Sevoflurane was also showed to have several other benefits: better antioxidative properties than propofol [33], reduced incidence of arrhythmias compared to desflurane [34], better preservation of cardiac function compared to control and to propofol [35,36] and to reduced NBP release and plasma protein A release when compared to propofol [37]. Isoflurane was reported to reduce troponin release when compared to propofol [38] but no differences were found when compared to sevoflurane [31,32]. Other authors on the contrary did not find differences in terms of troponin release when it was compared to a propofol group of patients [39]. Desflurane was reported to improve LSWI when compared to a propofol anesthetic regime, although no differences in troponin release were observed [40], and moreover was found to be less effective in reducing incidence of arrhythmias than sevoflurane [34]. Guarracino et al. observed a significant reduction in troponin release in a cohort of 57 patients anesthetized with desflurane [41]. Finally, remifentanil was found to have a preconditioning effect by lowering troponin release [42], however no differences were reported when was associated with sevoflurane and compared to propofol [30].

**Table 2:** **Studies including anaesthetics agents**

Mroziński et al. (*2014*) Anaesthesiol Intensive Ther, Poland [40] Prospective randomized open-label trial	Sixty OPCAB 28 Propofol 32 Desflurane	DES PP	Assessment of hemodynamic function and myocardial injury markers	DES group demonstrated improved stability, expressed as LVSWI; no differences in myocardial injury in between groups	No difference reported between DES and PP in major haemodynamic parameters, myocardial injury markers and the long-term outcome; DES might accelerate LVSWI recovery
Orriach et al. (*2013*) J Crit Care, Spain [25] Prospective randomized trial	Sixty OPCAB	SS and PP (intra-op and post op as postconditioning)	BNP Troponin release Need for inotropic drugs	SS group had reduced BNP, troponin release and number of inotropic drugs Compared to S-P and P-P groups	SS administration in OR and CICU, decreased troponin release compared with SS intra-op, but both were a better option to decrease troponin level when compared to PP
	20				
	Sevoflurane/Sevoflurane (S-S)				
	20 Sevoflurane/Propofol (S-P) 20 Propofol/Propofol (P-P)				
Wang et al. (*2013*) Scand Cardiovasc J, China [26] Prospective randomized controlled trial	Forty-eight OPCAB	SS	BNP	SS significantly decreased post-surgical troponin levels No significant differences in BNP level among groups	SS exerted significant myocardial protective effect; BNP could not predict myocardial protective effect of SS in OPCAB
	20 Sevoflurane		Troponin release		
	20 Control				
Suryaprakash et al. (*2013*) Ann Card Anaesth, India [28] Prospective randomized trial	One hundred thirty	SS	Troponin release	Changes in troponin levels at all time intervals were comparable in the three groups	No difference found in myocardial protection with SS or DES or PP
	nine OPCAB	DES			
	48 Sevoflurane	PP			
	52 Desflurane				
	39 Propofol				
Tempe et al. (*2011*) J Cardiothorac Vasc Anesth, India [38] Prospective randomized trial	Forty-five OPCAB	ISO	Troponin release	Troponin release in the PP group was significantly higher than the ISO group at 6 and 24 hours after surgery	ISO provided protection against myocardial damage by lowering levels of troponin-T
	Isoflurane	PP			
	Propofol				
Ballester et al. (*2011*) Eur J Anaesthesiol, Spain [33] Prospective controlled randomized trial	Thirty-eight OPCAB	SS	Markers of lipoperoxidation (F2-isoprostanes) and nitrosative stress (nitrates/nitrites) measured in coronary sinus blood	F2-isoprostanes concentrations were significant lower in the SS group at all different time point	SS showed better antioxidative properties than PP
	20 Sevoflurane	PP			
	18 Propofol				
Kim et al. (*2011*) Anaesth Intensive Care, Korea [29] Prospective randomized controlled trial	Ninety-four OPCAB	SS	CK MB and troponin release	No statistically differences in between groups in terms of CK-MB and troponin release at different end points	SS and PP had similar CK-MB and troponin values
	47 Sevoflurane	PP			
	47 Propofol	(both in a remifentanil based anesthesia)			
Hammerling et al. (*2010*) Ann Card Anaesth Canada [34] Prospective double blinded trial	Forty OPCAB	SS	Incidence of arrhythmias	Supraventricular tachycardia occurred only in the DES-group, AF was significantly more frequent in the DES group versus SEVO-group	SS found to be more advantageous than DES, as it was associated with less AF or supraventricular arrhythmias
	20 Sevoflurane	DES			
	20 Desflurane				
Xu (*2009*) J South Med Univ, China [42] Prospective controlled randomized trial	Twenty four OPCAB	REMI	Troponin release	Statistically significant reduction of troponin level in the REMI group	Troponin levels of REMI preconditioning group were markedly decreased after the operation in comparison with those of the control group
	12 Remifentanil				
	12 Control				
Drenger et al. (*2008*) Journal of Cardiothoracic and Vascular Anesthesia, Israel [21] Prospective controlled randomized trial	Twenty five OPCAB	IP induced with single 5 min LAD occlusion followed by 5 min reperfusion 1.6% ENF started 15 min before LAD occlusion	Myocardial metabolism	Lactate production in the ENF group decreased significantly compared with control and IP groups. Oxygen utilization in the control group was 44% higher than the other two groups Early recovery of anterior wall hypokinesis in both study groups	Application of methods such as IP or volatile anesthesia appeared to reduce the metabolic deficit
	8 Control				
	9 IP				
	8 Enflurane				
Hemmerling (*2008*) European Journal of Anaesthesiology, Canada [31] Prospective randomised trial	Fourty OPCAB	SS	Troponin/CK-MB	No differences in terms of enzymes release, heart contractility and haemodynamic values Extubation time was significantly shorter with SS compared to ISO	SS and ISO provided the same ischaemic cardio-protective effects; SEVO allowed a more rapid recovery from anaesthesia
	20 Sevoflurane	ISO	LVWM abnormalities time to extubation/respiratory functions haemodynamic parameters		
	20 Isoflurane				
Huseidzinović et al. (2007) Croat Med J, Croatia [35] Prospective randomised controlled trial	32 OPCABG	SS	Acceleration of aortic blood flow, CI, HR, mean arterial pressure, and central venous pressure at different time points	SS group showed better CI values at the beginning of ischemia and 15 minutes after ischemia; in the PP group acceleration decreased and remained lower 15 minutes after sternal closure while was increased in the SS group	Cardiac function was better preserved in patients with SS than with PP
	16 Sevoflurane				
	16 Control				
Venkatesh et al. (2007) Ann Card Anaesth, India [32] Prospective randomized	Forty OPCAB	SS	Haemodynamic effects amount of analgesia needed postoperative recovery	No differences identified in terms of haemodynamic parameters, depth of anesthesia, and quantity of agent needed; time of awakening and subsequent extubation were significantly less with SS	SS and ISO could both safely used in OPCAB; awakening and extubation time were significantly lower with SS.
	20 Isoflurane	ISO			
	20 Sevoflurne				
Lucchinetti et al. (*2007*) Anesthesiology, Switzerland [37] Prospective randomised trial	20 OPCAB	SS	Troponin, NBP and associate pregnancy-associated plasma protein A release Gene expression profile (atrial biopsies)	NPB and protein A were decrease in SS group; Echo showed preserved post-op LV function in SS group.	SS gene regulatory control of myocardial substrate metabolism predicted postoperative cardiac function in OPCAB patients
	10 Propofol	PP			
	10 Sevoflurane				
Guarracino et al. (*2006*) Journal of Cardiothoracic and Vascular Anesthesia, Italy [41] Prospective randomised trial	One hundred twelve	DES	Troponin release	Post-op peak troponin was significantly lower in DES group	Myocardial damage measured by cardiac troponin release could be reduced by DES during OPCAB
	OPCAB	PP			
	57 Desflurane	(in addition to opiate-based anesthesia)			
	55 Propofol				
Law-Koune (*2006*) J Cardiothorac Vasc Anesth, France [30] Prospective randomized trial	Eighteen OPCAB	SS-REMI	Troponin release	No difference in troponin release	Study did not support cardio-protective effects of SS
	9 Sevoflurane-remifentanil	PP-REMI			
	9 Propofol-remifentanil				
Bein et al. (*2005*) Anesth Analg, Germany [43] Prospective randomized trial	Fifty-two OPCAB (MIDCAB)	SS	Myocardial function	Myocardial performance index and early to atrial filling velocity ratio in the PP group deteriorated significantly whereas there was no change in the SS group	In patients undergoing MIDCAB surgery, SS preserved myocardial function better than PP
	26 Sevoflurane	PP			
	26 Propofol				
Kendall (*2004*) Anaesthesia, UK [39] Prospective randomized trial	Thirty OPCAB	PP	Troponin release	No significant difference in between groups	No support of ISO as cardioprotective agent was reported
	10 Propofol	ISO			
	10 Isoflurane	ISO/high thoracic epidural analgesia			
	10 Isoflurane and high thoracic epidural analgesia				
Conzen et al. (*2003*) Anesthesiology, Germany [27] Prospective randomized trial	Twenty OPCAB	SS	Troponin release	Troponin increased significantly more in the PP group rather than in the SS group	Patients receiving SS had less myocardial injury during the first 24 post-op hours than patients with PP
	10 Sevoflurane				
	10 Propofol				

*AF*: Atrial fibrillation; *BNP*: Brain natriuretic peptide *ONCAB*: On pump Coronary Artery By-pass Grafting; *OPCAB*: Off pump Coronary Artery By-pass Grafting; *DES*: Desflurane *IP*: Ischemic preconditioning; *LAD*: Left anterior descending artery; *LVWMS*: Left ventricle wall motion score; *MIDCAB*: Minimally invasive direct coronary artery bypass grafting; *PostC*: Postconditioning; *SS*: Sevoflurane.

### Alternative way to standard conditioning in OPCAB

There have been few reports on the use of adenosine as preconditioning agent in OPCAB. Forouzinna et al. [17] reported no differences between preconditioned and non preconditioned groups in terms of EF and troponin release. Li et al. [6] randomized a small number of patients to on and off-pump to be preconditioned with the use of hyperbaric oxygen (HBO). Patients in the preconditioning group underwent HBO for 70 min/daily for 5 consecutive days before surgery. Preconditioning with HBO resulted in both cerebral and cardiac protective effects as determined by biochemical markers of neuronal and myocardial injury and clinical outcomes in patients undergoing ONCAB while no benefits were observed in the OPCAB group. Matsumoto et al. [24] reviewed a cohort of 48 OPCAB patients. Among them, the subgroup treated with IP plus allopurinol and nicorandil had an improved post-ischemic functional recovery after surgery.

Myocardial conditioning in cardiac surgery can be achieved in different ways. The most used are central or remote preconditioning and volatile anesthetics, while adenosine or other pharmacological agents less frequently used. Ischemic conditioning was first reported in 1986 [4] and conditioning by volatile anesthetic with halothane in 1976 [44]. Although different, both techniques share probably some common mechanisms of action and, most importantly, can be used simultaneously. Both have been frequently utilized in ONCAB [45], but with regard of OPCAB surgery no large trials have been published so far evaluating their effect on troponin [19] or clinical outcomes. Moreover, according to clinical trial.gov [46], while there are different trials in different phases, registered and ongoing, investigating the effect of RIPC in ONCABG (isolated or plus/minus valve surgery) the only trial aiming to compare the effect of RIPC on ON and OFF-pump CABG is the RIP-CON study, expected to report in June 2015 [47]. Small trials are present in the literature, focusing on the effect of RIPC on different subsystem outcomes. Joung et al. [16] randomised 70 OPCAB patients to RIPC and control with cognitive function as primary end points. Hong et al. reported no differences in terms of troponin release in OPCAB after RIPC [19]. Same Authors [18], however, studied the effect of RIPC and PostC together and they observed, this time, a reduction of troponin release in a series of OPCAB. All the other trials [13,14,17,20-22,48] carried out in OPCAB with conditioning used ‘central preconditioning’, hence eliciting protection by cycles of occlusion/reperfusion at the level of targeted vessel. All of them led to different and non-homogeneous results. Central remote ischemic preconditioning has been, however, fairly dismissed in on pump cardiac surgery and during interventional cardiology procedure for its impracticality and its high level of invasiveness [2,3,49]. It is important to stress that some of the RIPC and central IP trials did not state the type of anesthesia used [13,23], or deliberately used propofol as anesthetic maintenance [12,16-18,20], while very few did not use it [19,21,22]. Recently it has been reported that propofol may inhibit preconditioning effect, and large trials have intentionally avoided it use [50].

Since the first report in 1976 [44] volatile anaesthetics have been frequently used as conditioning techniques in ONCAB. Two large meta-analyses from 2006 [51,52] have suggested that there was a significant difference in in patients who received volatile agents in terms of reduced troponin release, improved CI, less use of inotropes and reduced need of mechanical ventilation. However they did not point out any differences with regards of mortality, LOS and myocardial infarction rate. Moreover a relatively recent review [53] has reported that sevoflurane had beneficial effects only in naïve patients with no previous exposures to episode of angina. Although there is sufficient evidence to support the use of volatile agents during cardiac surgery and taking into account their feasibility to be used as pre, per and post conditioning with the potential to prolong the protective effect [25], no large trials have been set in place in order to identify potential benefits in patients undergoing OPCAB. Up till now there are 17 trials comparing different volatile anesthetics *vs* propofol or *vs* control or volatiles among themself but the vast majority of them were underpowered and led to conflictive results (Table 2). Sevoflurane is proven to reduce troponin level after on-pump surgery but its effect is still unclear in the off-pump scenario. With regard to OPCAB, sevoflurane was found to preserve cardiac function in three studies [35,37,54], to reduce troponin release in two studies [26,27], to have antioxidant properties in one [33] and to prevent incidence of arrhythmias in one [34]. On the contrary sevoflurane did not show any better troponin reduction when compared to other agents in four studies [28-31]. Only Orriach et al. used sevoflurane during and after the operation as post-conditioning and reported a significant reduction in troponin release [25].

## Conclusions

Although OPCAB is thought to reduce the extent of general ischemia, ischemic cardiac insult can be found in up to 10% of the patients [12]. Data coming from the largest pooled analysis on mainly on-pump experience reported a trend toward a consistent reduction of almost a half of MI in RIPC groups [55]; hence there may be the theoretical potential to translate the same advantage to off-pump patients. However, up till now there are no strong evidences supporting the use of ischemic preconditioning, either central or remote, in OPCAB patients.

In terms of volatile anaesthetics, taking into account all the trials, we can conclude here that has not yet been demonstrated if they can reduce troponin level after off-pump surgery. Other forms of conditioning used in OPCAB such as adenosine/HBO/pharmacological have been used and some times in association with IP, but led to different conclusions. Conditioning, either elicited by ischemia/reperfusion or by volatile agents, may theoretically be a valid method to increase cardiac protection in off-pump surgery, but further trials are definitely needed.

## Abbreviations

AF: Atrial fibrillation
BNP: Brain natriuretic peptide
CABG: Coronary Artery By-pass Grafting
DES: Desflurane
HBO: Hyperbaric oxygen
IP: Ischemic preconditioning
I/R: Ischemia reperfusion
LAD: Left anterior descending artery
LVWMS: Left ventricle wall motion score
MIDCAB: Minimally invasive direct coronary artery bypass grafting
ONCAB: On-pump CABG
OPCAB: Off-pump CABG
phiL /phiT: Ratio of longitudinal to transverse conduction velocity
PostC: Postconditioning
RIPC: Ischemic remote preconditioning
SVI: Stroke volume index
SS: Sevoflurane
TIVA: Total intra-venous anesthesia
